# Recent advances of CRISPR-based genome editing for enhancing staple crops

**DOI:** 10.3389/fpls.2024.1478398

**Published:** 2024-09-23

**Authors:** Feng Chen, Lu Chen, Zhao Yan, Jingyuan Xu, Luoluo Feng, Na He, Mingli Guo, Jiaxiong Zhao, Zhijun Chen, Huiqi Chen, Gengzhen Yao, Chunping Liu

**Affiliations:** ^1^ School of Biology and Food Engineering, Changshu Institute of Technology, Changshu, Suzhou, Jiangsu, China; ^2^ Pharma Technology A/S, Køge, Denmark; ^3^ State Key Laboratory of Dampness Syndrome of Chinese Medicine, The Second Affiliated Hospital of Guangzhou University of Chinese Medicine, Guangzhou, Guangdong, China; ^4^ Second Clinical Medical College, Guangzhou University of Chinese Medicine, Guangzhou, China; ^5^ Guangdong-Hong Kong-Macau Joint Lab on Chinese Medicine and Immune Disease Research, Guangzhou, Guangdong, China

**Keywords:** climate change, CRISPR/Cas system, genetic modifications, abiotic stress, global food security, sustainable agricultural

## Abstract

An increasing population, climate change, and diminishing natural resources present severe threats to global food security, with traditional breeding and genetic engineering methods often falling short in addressing these rapidly evolving challenges. CRISPR/Cas systems have emerged as revolutionary tools for precise genetic modifications in crops, offering significant advancements in resilience, yield, and nutritional value, particularly in staple crops like rice and maize. This review highlights the transformative potential of CRISPR/Cas technology, emphasizing recent innovations such as prime and base editing, and the development of novel CRISPR-associated proteins, which have significantly improved the specificity, efficiency, and scope of genome editing in agriculture. These advancements enable targeted genetic modifications that enhance tolerance to abiotic stresses as well as biotic stresses. Additionally, CRISPR/Cas plays a crucial role in improving crop yield and quality by enhancing photosynthetic efficiency, nutrient uptake, and resistance to lodging, while also improving taste, texture, shelf life, and nutritional content through biofortification. Despite challenges such as off-target effects, the need for more efficient delivery methods, and ethical and regulatory concerns, the review underscores the importance of CRISPR/Cas in addressing global food security and sustainability challenges. It calls for continued research and integration of CRISPR with other emerging technologies like nanotechnology, synthetic biology, and machine learning to fully realize its potential in developing resilient, productive, and sustainable agricultural systems.

## Introduction

The ever-increasing global population and the consequent demand for food have placed immense pressure on agricultural systems worldwide. This challenge is compounded by the escalating impacts of climate change, which include extreme weather events, shifting pest and disease patterns, and declining arable land (Bibi and Rahman, 2023). These changes threaten crop yields and disrupt agricultural stability, making the task of ensuring global food security increasingly daunting. Traditional breeding methods, while having significantly contributed to past agricultural advancements, are often too slow to respond to these rapid environmental changes, while genetic engineering has faced issues of precision and public acceptance (Afzal et al., 2023; Ambika et al., 2024).

Staple crops such as rice, wheat, maize, and soybeans are the backbone of global food security, providing the primary source of calories for a large portion of the world’s population (Morrow et al., 2023). These crops are crucial not only for direct human consumption but also for animal feed and industrial uses. However, the productivity and resilience of these staple crops are increasingly threatened by climate change, pests, and diseases. Improving the yield, nutritional content, and stress tolerance of staple crops is therefore essential for ensuring food security, particularly in the face of a growing global population and diminishing arable land.

The evolution of agricultural technology from selective breeding to sophisticated genetic tools underscores our ongoing efforts to address these challenges. CRISPR/Cas technology as a revolutionary genome-editing tool has emerged as a game-changer in agricultural biotechnology (Muha-Ud-Din et al., 2024). CRISPR/Cas systems, a groundbreaking tool for targeted genome editing, have revolutionized both basic and applied research in agriculture. Originally derived from the adaptive immune systems of bacteria and archaea, the CRISPR (Clustered Regularly Interspaced Short Palindromic Repeats) mechanism uses a guide RNA (gRNA) to direct the Cas (CRISPR-associated) nuclease to a specific DNA sequence, where it creates a precise double-strand break. This break is subsequently repaired by the cell’s natural DNA repair mechanisms, allowing for targeted modifications to the genome (Ghosh and Chatterjee, 2024). Unlike earlier genome editing tools like Zinc Finger Nucleases (ZFNs) and Transcription Activator-Like Effector Nucleases (TALENs), CRISPR/Cas systems are easier to design, more efficient, and less expensive, making them highly accessible for a wide range of applications in crop improvement (Ghoshal, 2024). The discovery of the CRISPR/Cas system as a genome editing tool was not just about identifying it in bacteria, but also about understanding how it could be harnessed and refined for use in more complex organisms. Early research clarified the critical roles of crRNA (CRISPR RNA) and tracrRNA (trans-activating crRNA) in guiding the Cas9 protein for precise DNA cleavage, which was pivotal in developing CRISPR/Cas into a versatile genome editing tool (Jung et al., 2024). The mechanism of action begins with the formation of an RNA-DNA hybrid, where the guide RNA binds to the target DNA sequence, directing the Cas9 protein to the specific genomic site. Once there, Cas9 introduces a double-strand break, which is then repaired by the cell’s natural DNA repair pathways—either non-homologous end joining (NHEJ) or homology-directed repair (HDR) (Yuan et al., 2024). This detailed understanding of the CRISPR/Cas mechanism underscores its effectiveness in enabling precise and efficient genome modifications, making it a cornerstone technology for advancing crop traits and addressing global challenges such as food insecurity and climate change (Raza et al., 2024).

CRISPR technology has emerged as a transformative tool, allowing for the rapid development of crop varieties with enhanced traits such as improved resistance to biotic and abiotic stresses, increased nutritional value, and greater yield potential (Verma et al., 2023). Moreover, unlike traditional genetic modification techniques, CRISPR/Cas systems enhance agricultural productivity and sustainability through their simplicity, adaptability, cost-effectiveness, and publicly acceptable approach due to its ability to make precise alterations without introducing foreign DNA (Ali et al., 2023). Recent advancements, such as prime editing and base editing, have further refined the precision and scope of genome editing, enabling more complex genetic enhancements with fewer off-target effects (Naeem and Alkhnbashi, 2023; Saber Sichani et al., 2023). These innovations are paving the way for next-generation crops that can thrive in changing environmental conditions and meet the nutritional needs of a growing population.

This review aims to provide a comprehensive overview of CRISPR/Cas technology in enhancing crop resilience and productivity of staple grains amidst climate challenges. By exploring the latest research and technological advancements, this article highlights the transformative potential of CRISPR/Cas systems in modern agriculture. It would provide comprehensive insights for understanding current innovations and identifying strategic directions for future research and development, ultimately contributing to global food security and sustainable agricultural practices.

## CRISPR/Cas technological innovations and advancements

**Figure 1:**
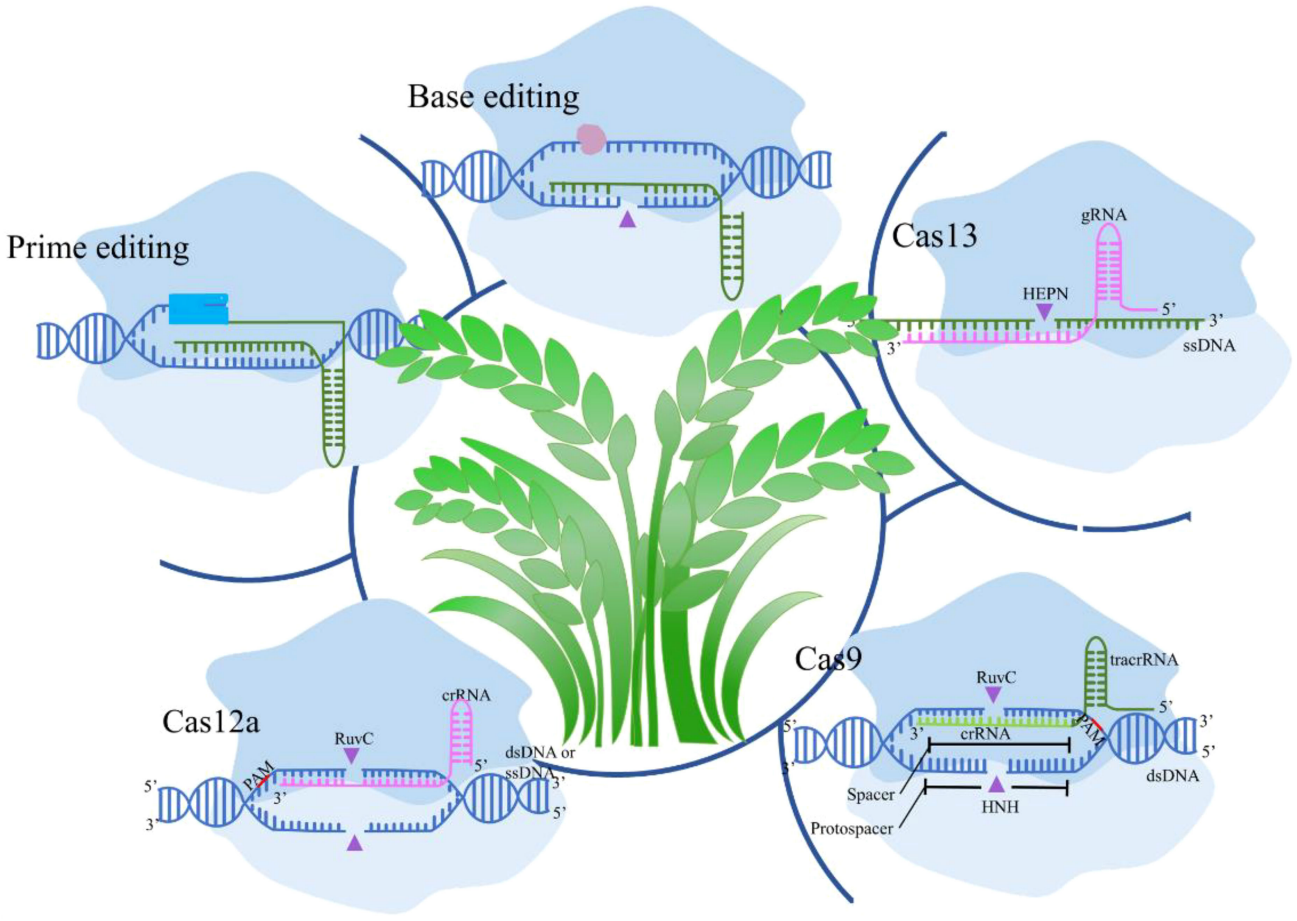
Diverse CRISPR/Cas systems mediated biotechnologies in crops. Cas9 in crops introduces double-strand breaks (DSBs) for targeted gene knockouts or insertions; Cas12a creates staggered DNA cuts and allows for multiplexed editing; Cas13 targets RNA for post-transcriptional regulation without altering the genome; Base Editing enables precise single-nucleotide changes without DSBs; and Prime Editing allows for small insertions, deletions, and base conversions with high precision.

Recent advancements in CRISPR technology have significantly enhanced the specificity and efficiency of genome editing, crucial for agricultural applications (**Figure 1**). Innovations like prime editing and base editing represent groundbreaking developments in precise genetic alterations. Prime editing combines CRISPR-Cas9 with a reverse transcriptase which has the potential to correct up to 89% of known genetic variants, enabling direct editing of target DNA sequences (Chen and Liu, 2023). Studies have demonstrated its effectiveness in enhancing disease resistance in rice by correcting specific point mutations without causing double-strand breaks (Gupta et al., 2023). Conversely, base editing facilitates the direct and irreversible conversion of one DNA base into another, increasing the precision of point mutations (Pfeiffer and Stafforst, 2023). Applications include altering flavor profiles in pea and tomatoes and improving cold tolerance in soybeans by modifying genes responsible for fatty acid desaturation and cold response pathways (Nizampatnam et al., 2024). Novel CRISPR-associated proteins, such as Cas12 and Cas13, expand the toolkit available for agricultural biotechnology. Cas12 offers advantages for multiplex editing, allowing simultaneous manipulation of multiple traits, for example, facilitate several disease resistance genes in soybeans (Sun et al., 2024). Cas13d offers a particularly robust solution for multiplex RNA virus interference in potato crops, making it a valuable asset in the ongoing efforts to enhance agricultural productivity and sustainability (Zhan et al., 2023).

**Figure 2:**
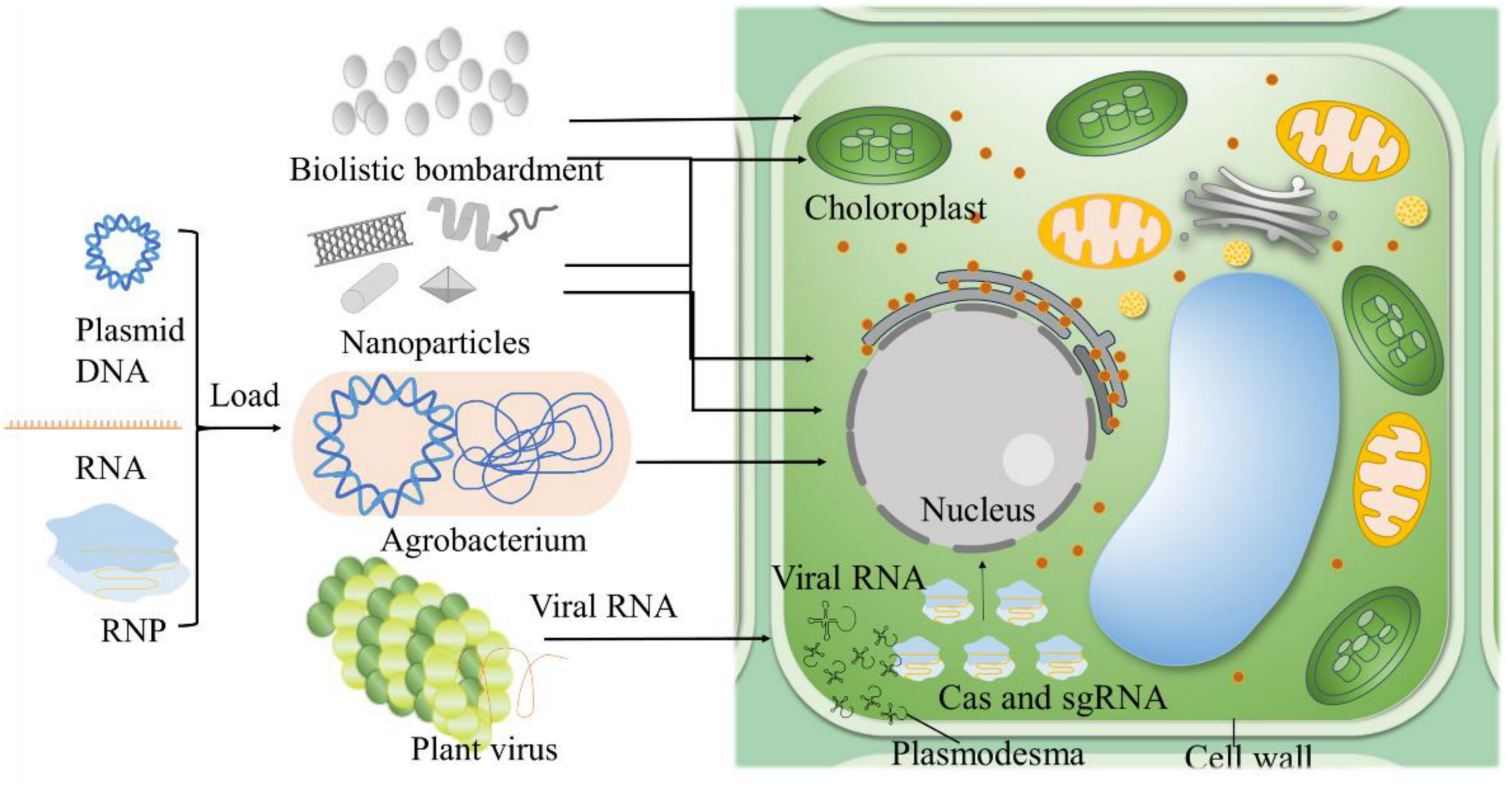
Strategies for CRIPSR/Cas delivery. Agrobacterium-mediated transformation exploits the bacterium's ability to transfer genetic material into plant genomes. Nanoparticles serve as carriers for CRISPR/Cas components, easily passing through plant cell walls. Biolistic bombardment which physically shoots DNA, RNA, or RNP-coated particles into plant tissues, useful for targeting chloroplasts and difficult-to-transform species. Virus-mediated delivery incorporates CRISPR/Cas into plant viruses, enabling systemic delivery across the plant. Partial figures are modified from previous publications (Zhu et al., 2020).

Efficient delivery of CRISPR components is essential for successful genome editing in plants (**Figure 2**). Recent methodologies include nanoparticle-mediated delivery, which protects CRISPR components from degradation and enhances cellular uptake, significantly improving trait enhancement in maize (Chakraborty et al., 2023; Yau et al., 2024). Viral vectors, leveraging natural viral infection mechanisms, have shown improved efficiency and safety in transient expression projects, such as inducing virus resistance traits in tobacco and tomato (Jogam et al., 2023; Wang et al., 2024e). The ribonucleoprotein (RNP) complex delivery method delivers CRISPR components directly as proteins and RNA, reducing off-target effects. This method has been effective in crops like wheat for disease resistance and yield enhancement (Poddar et al., 2023). Enhanced computational tools for precise guide RNA design and the development of high-fidelity Cas variants exhibit reduced off-target activity (Zhang et al., 2023b). High-fidelity Cas9 variants have been used in wheat to reduce unintended mutations while enhancing drought tolerance (Poddar et al., 2023).These advancements not only improve the safety of genetic edits but also broaden CRISPR’s applicability in developing climate-resilient crops. The continuous refinement of CRISPR technologies, including the development of novel delivery methods and editing techniques is paving the way for transformative advances in agriculture in **Table 1**. By increasing the precision and efficiency of these tools, researchers are expanding the potential applications of CRISPR, facilitating the creation of more resilient, productive, and sustainable agricultural systems.

**Table 1:** CRISPR/Cas technological innovations and advancements.

	Types	Mechanism	Advantages	Stable Crops	Traits	References
Innovation	CRISPR/Cas9	Use guide RNA to target and Cas9 to cut DNA	High efficiency, broad applicability	Barley	Coleoptile length increasing	(Cheng et al., 2023)
	Base Editing	Convert one DNA base to another without double-strand breaks	High precision, avoids double-strand breaks	Rice	Glyphosate resistance	(Sony et al., 2023)
	Prime Editing	Use CRISPR-Cas9 with a reverse transcriptase to make precise edits	Versatile, corrects point mutations	Rice; Peanut; Chickpea	Restoration of GFP activity	(Biswas et al., 2022)
	CRISPR/Cas12a	Use guide RNA to target and Cas12a to cut DNA	Multiplex editing, higher specificity in some contexts	Rice; Maize	Root-knot nematode resistance; Chlorotic mottle virus resistance	(Lei et al., 2023; Zhou et al., 2023b)
	CRISPR/Cas13	Target RNA instead of DNA	RNA targeting, potential for viral resistance	Potato	Multiplex viruses resistance	(Zhan et al., 2023)
Delivery Methods	Nanoparticle-Mediated Delivery	Use nanoparticles to deliver CRISPR components	High protection, enhanced uptake	Maize;	Trait enhancement;	(Nagy et al., 2023)
	Viral Vectors	Employ viruses to deliver CRISPR components	Utilizes natural infection mechanisms	Cassava	Precision breeding	(Tuo et al., 2023)
	Ribonucleoprotein (RNP) Complexes	Direct delivery of CRISPR-Cas9 protein and guide RNA as a complex	DNA-free method, reduces potential off-target effects	Potato; Wheat	Color change; Diversity production accelerating	(Poddar et al., 2023; Wulff-Vester et al., 2024)
	Agrobacterium-Mediated Transformation	Use Agrobacterium to transfer CRISPR components into plant cells	Effective for stable transformations	Rice	Agronomic trait improving	(Tang et al., 2023)
	Biolistic Particle Delivery	Use high-velocity particles to deliver CRISPR components into cells	Versatile, can deliver to a wide range of species	Millet	Genetic modifications; stress tolerance improving	(Ghosh, 2024)
Off-target Mitigation	Enhanced Guide RNA Design	Optimize guide RNA sequences for specificity	Reduces off-target effects, increases accuracy	Wheat; maize	Precision editing; stress tolerance improving	(Abeuova et al., 2023; Karmacharya et al., 2023)
	High-Fidelity Cas9 Variants	Engineer Cas9 proteins with reduced off-target activity	Increases efficient, reduces unintended mutations	Barley; Wheat	Targeted trait enhancements	(Lawrenson et al., 2024)
	Computational Tools	Use software to predict and minimize off-target effects	Improves design accuracy, reduces experimental time	Maize; Wheat	Yield improving	(Gaillochet et al., 2023)
	Use of Shortened Guide RNAs	Shorten versions of guide RNAs to improve targeting precision	Decreases off-target activity, maintains efficiency	Sorghum	Precision gene editing	(Lee et al., 2023)
	Paired Nickases	Use two nicks instead of a double-strand break to reduce off-target effects	Reduces off-target activity, increases precision	Potato	Trait improvement	(Mali and Zinta, 2024)

## Strategic applications of CRISPR/Cas in enhancing staple crop resilience

**Figure 3:**
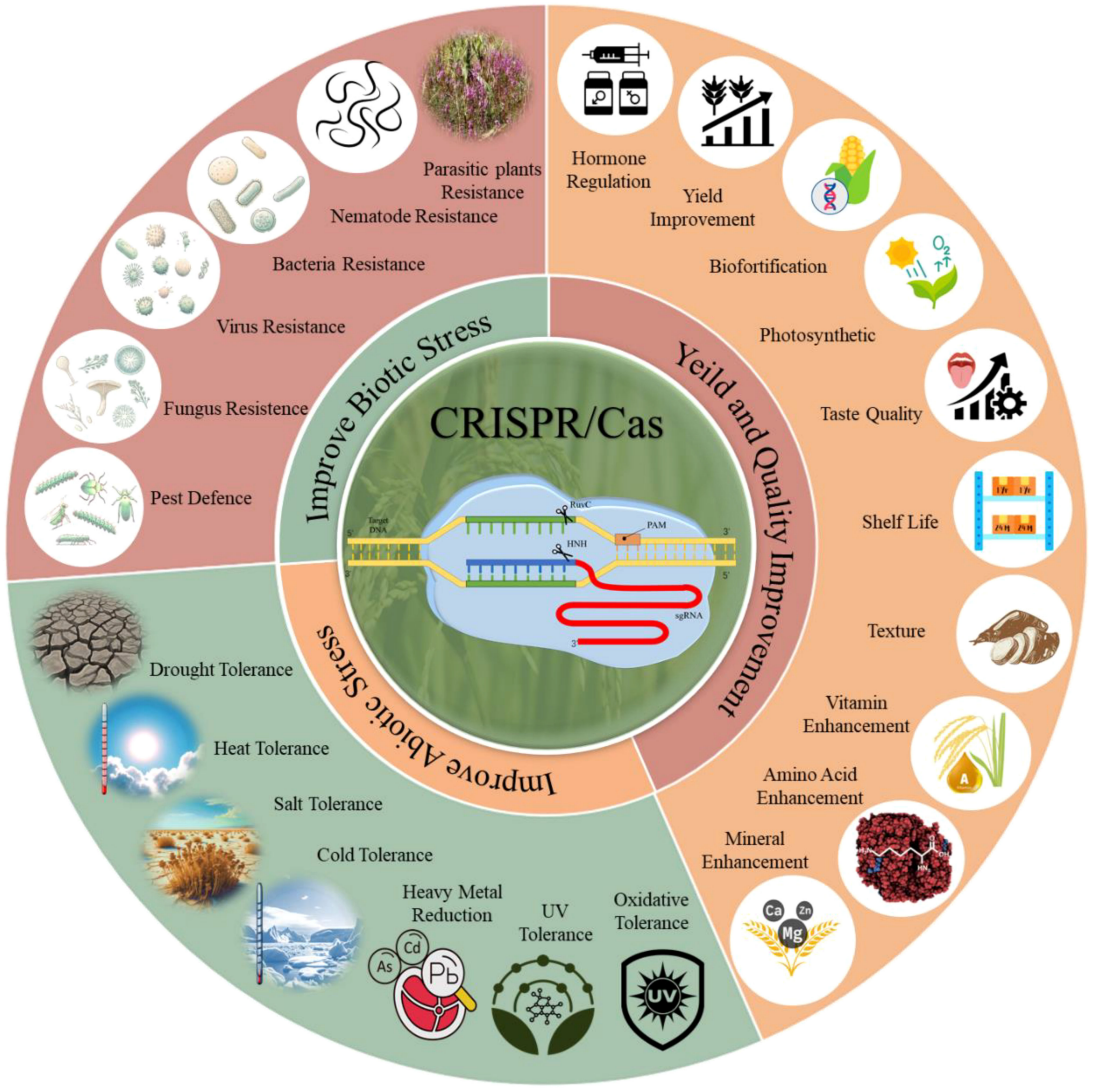
CRISPR/Cas genome editing is a powerful tool with immense potential for crop improvement. It enables precise modifications of individual genes to enhance plant tolerance to biotic and abiotic stresses, as well as yield and quality improvement. CRISPR/Cas has been used to develop resistance against various biotic stresses, including bacteria, viruses, fungi, pests, nematodes, and parasitic plants, and to improve resilience to abiotic stresses like drought, heat, salt, cold, heavy metals, UV exposure, and oxidative stress. Additionally, CRISPR/Cas enhances crop yield and quality by regulating hormone production, increasing photosynthetic efficiency, supporting biofortification, and improving shelf life, texture, and taste.

The application of CRISPR/Cas technology in agriculture holds immense potential for improving the resilience of grain crops against various abiotic and biotic stresses (Yadav et al., 2023). This section explores how recent advancements in CRISPR/Cas technology have enhanced grain crop tolerance to these stresses, thereby supporting sustainable agricultural productivity in the face of climate change and other environmental challenges. CRISPR/Cas genome editing has become a mature tool for improving crop growth, development, and stress responses, as illustrated in **Figure 3**. In this context, we reviewed recent advances in CRISPR-mediated crop enhancement under abiotic and biotic stresses and improvements in various growth-related traits.

### Enhancing abiotic stress tolerance

Abiotic stresses significantly reduce agricultural productivity and crop yields, but CRISPR/Cas technology provides precise tools to enhance grain crop tolerance by targeting specific stress response genes (**Table 2**).

**Table 2:** CRISPR/Cas technological innovations and advancements in stable crops.

Application	Hazardous factors	Innovation	Stable Crops	Targeted genes	Applications in Stable Crops	References.
Enhancing Abiotic stress Tolerance	Drought	CRISPR/Cas9	Maize	*ZmHDT103*	Enhanced drought resistance	(Wang et al., 2024f)
			Wheat	*TaRR12*		(Li et al., 2024a)
			Rice	*OsPUB7*		(Kim et al., 2023)
			Potato	*StDRO2*		(Zhao et al., 2024)
	Heat	CRISPR/Cas12	Rice	*OsDEP1,OsROCs*	Enhanced heat resistance	(Malzahn et al., 2019)
		CRISPR/Cas9	Rice	*OsGER4*		(Nguyen et al., 2023)
			Wheat	*TaHSFA1*		(Wang et al., 2023)
			Soybean	*GmHsp90A2*		(Jianing et al., 2022)
			Maize	*ZmHSPs*		(Li et al., 2024b)
	Salt	CRISPR/Cas9	soybean	*GmNHL1*	Enhanced salinity tolerance	(Liu et al., 2023b)
			Barley	*HvGSK1.1*		(Kloc et al., 2024)
			Rice	*OsTPP3*	Improved Salt resistance	(Ye et al., 2023)
			Wheat	*TaHKT1;5*		(Wang et al., 2024b)
	Cold	CRISPR/Cas9	Rice	*bHLH57*	Increased grain yield under normal and chilling conditions	(Zhang et al., 2023c)
			Maize	*ZmG6PDH1*	Enhanced cold resistance	(Li et al., 2023a)
			Potato	*VInv*		(Yasmeen et al., 2022)
			Wheat	*TaPGK*		(Zhang et al., 2023e)
	Heavy Metal	CRISPR/Cas9	Rice	*K5.2*	Increased Ca accumulation	(Wang et al., 2024c)
				*OsNIP3*	Reduced arsenic accumulation	(Xu et al., 2024)
				*OsLCD*	Generated low-cadmium germplasms	(Chen et al., 2023)
				*OsMYB84*	Modulated copper uptake and transport	(Ding et al., 2024)
				*NRAMP1, FRO2*	Improved Fe uptake from the soil	(Krishna et al., 2023)
				*OsPDR7, OsZIP9*	Enhanced zinc accumulation	(Lu et al., 2023)
			Wheat	*TaIPK1*	Improved iron and zinc accumulation	(Ibrahim et al., 2022)
	UV Radiation	CRISPR/Cas9	Rice	*OsCOP1*,	Improved UV protection	(Hu et al., 2024)
	Oxidative	CRISPR/Cas9	Rice	*OsCAT2*	Alleviates the oxidative stress by scavenging ROS	(Shen et al., 2024)
Enhancing Biotic stress Tolerance	Virus	CRISPR/Cas13	Potato	*PVY, PVS, PVX or PLRV*,	Reduced viral infections;	(Zhan et al., 2023)
		CRISPR/Cas9	Maize	*Zmpdrp1*	Reduced robust virus	(Xie et al., 2024)
			Rice	*OsCPR5.1*	Yellow mottle virus resistance	(Arra et al., 2024)
			Potato	*SlDCL2b*	Spindle tuber viroid resistance	(Tiwari et al., 2022)
			Cassave	*MeRPPL1*	Resistance to geminivirus	(Ramulifho and Rey, 2024)
		CRISPR/Cas12a	Maize	*MCMV*	Reduced viral infections	(Lei et al., 2023)
	Bacterial	CRISPR/Cas9	Rice	*OsPUB9*	Resistance to bacterial leaf blight	(Kim et al., 2024)
			Potato	*StNRL1*	resistance to late blight and susceptibility to early blight	(Norouzi et al., 2024)
	Fungi	CRISPR/Cas9	Soybean	*Glyma05g29080*	Resistance to white mold	(Zhang et al., 2022a)
			Wheat	*TaCIPK14*	Resistance to stripe rust	(He et al., 2023)
			Maize	*ZmAGO18b*	Resistance to southern leaf blight	(Dai et al., 2023)
			Rice	*Pi21, OsSULTR3;6*	Resistance to rice blast	(Yang et al., 2023a)
			Barley	*BnHva22c*	Reduced fungal pathogen, susceptibility	(Ye et al., 2024)
	Pest	CRISPR/Cas9	Maize	*Cry1F*	Improved pest resistance	(Kumari et al., 2024)
			Rice	*OsWRKY71, Bph15*	Resistance against brown plant hopper	(Li et al., 2023b)
			Soybean	*GmUGT*	Resistance against leaf-chewing Insects	(Zhang et al., 2022b)
	Nematode	CRISPR/Cas9	Rice	*OsHPP04*	Resistance to rice root-knot nematode	(Huang et al., 2023)
			Soybean	*GmSNAP11, α-SNAP*	Resistance to soybean cyst nematode	(Shaibu et al., 2022; Usovsky et al., 2023)
	Parasitic Plants	CRISPR/Cas9	Rice	*PR10/Bet v1-like protein gene*	Resistance against *Meloidogyne graminicola.*	(Li et al., 2022b)
			Sorghum	*CCD*	Control the germination of a parasitic weed (*Striga* spp.)	(Hao et al., 2023)

### Drought stress

Staple grain crops such as rice, wheat, and maize are particularly vulnerable to water scarcity, which poses a major challenge to food security. CRISPR/Cas technology allows precise genetic modifications to improve drought tolerance by targeting genes that regulate water use efficiency and osmotic balance (Shelake et al., 2022). A notable breakthrough in this area is the modification of the *ZmHDT103* gene, a key component of the abscisic acid (ABA) signaling pathway, which has been shown to improve drought tolerance in maize by enhancing the plant’s ability to withstand water scarcity without compromising growth and yield under non-stress conditions (Wang et al., 2024f). Another promising application of CRISPR technology in combating drought stress involves engineering the *TaRPK1* gene in wheat to enhance water absorption (Abdul Rahim et al., 2024). By targeting genes that influence root growth patterns and depth, CRISPR/Cas has been used to develop plants with deeper root systems capable of accessing water from further soil layers. Another innovative approach involves using CRISPR/Cas to manipulate *Sal1* genes to improve the production of osmoprotectants, such as proline, which has been shown to enhance drought resistance in wheat by allowing plants to withstand dry periods more effectively through precise modulation of gene expression (Mohr et al., 2022).

### Heat stress

Elevated temperatures can impair plant growth, reduce photosynthetic efficiency, and ultimately decrease crop yields, leading to a focus on using CRISPR/Cas technology to modify heat shock factors (*HSFs*) and heat shock proteins (*HSPs*) to enhance heat tolerance (Younas et al., 2024). These proteins protect cells from heat damage by refolding denatured proteins and preventing the aggregation of unfolded proteins. For instance, enhancing *HsfA1b* expression in wheat improves heat tolerance, maintaining growth and yields under high temperatures (Tian et al., 2020). Researchers are also editing genes involved in the synthesis of protective osmolytes and antioxidants to mitigate oxidative stress caused by high temperatures (Yadav et al., 2023). Furthermore, integrating CRISPR/Cas with other biotechnological tools enhances the robustness of crops against heat stress. For instance, gene drives engineered through CRISPR technology can spread heat tolerance traits rapidly through plant populations, potentially transforming the resilience of an entire crop in a few generations (Mohan et al., 2022).

### Salt stress

Salt stress, an increasing agricultural concern particularly in regions affected by soil salinization due to improper irrigation practices and rising sea levels, is being addressed by CRISPR/Cas technology through the enhancement of salt tolerance by targeting genes that regulate ion homeostasis and osmotic balance (Hualpa-Ramirez et al., 2024). One strategy involves modifying transporters involved in sodium uptake and compartmentalization. For example, editing genes like knocking out *AKT1*, *WRKYs* reduces sodium accumulation, thereby improving salt tolerance and maintain better growth and yield in soybean and barley (Price, 2022; Feng et al., 2023). Additionally, by upregulating the *DREB2A* transcription factor has successfully enhanced salt tolerance in various crops, including soybeans and rice (Farhat et al., 2019; Feng et al., 2023). By fine-tuning the expression of these transcription factors, crops can activate comprehensive stress response pathways that confer enhanced tolerance to saline conditions.

### Cold stress

CRISPR/Cas technology facilitates targeted genomic edits to confer cold tolerance by targeting the CBF (C-repeat Binding Factor) pathway, a well-documented regulatory mechanism in plants that enhances cold tolerance. CBF transcription factors activate a suite of genes that confer cold tolerance by enhancing the plant’s cellular machinery to cope with freezing stress (Perez-Garcia et al., 2023). CRISPR/Cas has been used to increase the expression of fatty acid desaturase genes, leading to changes in membrane lipid composition that better support cellular processes during cold exposure. For instance, studies have demonstrated that OsKASI-2 is required for increasing unsaturated fatty acids in rice membranes via CRISPR/Cas to improve cold tolerance (Zhang et al., 2024b). Another innovative approach involves using CRISPR/Cas for epigenetic modifications that influence gene expression related to cold stress (Jogam et al., 2022). This method offers a flexible approach to crop improvement that can be adjusted as environmental conditions change.

### Heavy metal stress

Heavy metals such as cadmium (Cd), arsenic (As), and lead (Pb) are toxic to plants, causing stunted growth and reduced yields, but CRISPR/Cas technology can be used to enhance plant tolerance to heavy metals by modifying genes involved in metal transport and detoxification. For instance, editing the *OsLCD* gene in rice reduces cadmium uptake, thereby increasing cadmium tolerance and reducing its accumulation in the edible parts of the plant (Chen et al., 2023). Similarly, targeting genes like *Lsi1* and *Lsi2* in rice can decrease arsenic accumulation in plant tissues, thereby improving tolerance to arsenic-contaminated soils (Xu et al., 2024). Furthermore, targeting the *ZmHMA3* gene in maize has shown to increase zinc tolerance by enhancing the plant’s ability to compartmentalize and detoxify these metals (Lv et al., 2023). These advancements contribute to the development of crops capable of growing in contaminated soils and producing safer food products.

### UV radiation stress

CRISPR/Cas technology offers a promising solution to the significant threat of UV radiation to crop health by enabling precise genetic modifications that enhance tolerance to DNA damage, oxidative stress, and impaired photosynthesis. Targeting the *OsCOP1* gene has demonstrated potential in improving UV tolerance in rice, enhancing their resistance to UV-B radiation (Hu et al., 2024). By boosting the plant’s protective mechanisms against UV damage, CRISPR/Cas technology can help develop crops that maintain productivity and growth under high UV exposure.

### Oxidative stress

Oxidative stress results from the accumulation of reactive oxygen species (ROS) under various stress conditions. CRISPR/Cas technology provides a means to enhance oxidative stress tolerance in crops by targeting genes involved in ROS detoxification and antioxidant defense mechanisms. For example, Editing the CAT (catalase) gene family, particularly *OsCAT3*, which is crucial for detoxifying superoxide radicals and hydrogen peroxide, can enhance rice’s ability to mitigate oxidative damage (Jiang et al., 2023). Additionally, targeting regulatory genes such as zinc finger proteins, which modulate the expression of multiple antioxidant genes, can offer a comprehensive approach to improving oxidative stress resilience in crops (Qu et al., 2024).

### Improving biotic stress resistance

Biotic stresses threaten crop health and productivity, and CRISPR/Cas technology enables precise genetic modifications to enhance crop resistance.

### Viruses stress

CRISPR/Cas systems, particularly Cas13 have shown targeting and degrading the RNA genomes of RNA viruses, preventing their replication within the host plant (Sarkar et al., 2024). This approach has been effectively demonstrated in crops such as potato, where Cas13 was engineered to target and cleave the RNA of sweet potato virus disease (Zhan et al., 2023). Researchers have expanded the capabilities of CRISPR/Cas systems in viral defense by using them not only to target pathogens directly but also to modify the host plant’s genome to enhance its natural virus defense mechanisms (Uranga and Daròs, 2023). For instance, in crops like wheat and rice, CRISPR/Cas9 has been employed to knock out susceptibility genes such as *TaPDIL5* or *OsDjA2* and *OsERF* that facilitate viral infection, thus providing broad-spectrum virus resistance (Kan et al., 2022; Távora et al., 2022). Additionally, CRISPR/Cas technology was also employed to knock out *ZmPDRP1* in maize, revealing that the loss of this gene significantly reduced the ability of sugarcane mosaic virus (SCMV) to replicate and spread within the plant (Xie et al., 2024). This highlights the utility of CRISPR/Cas not only for plant trait improvement but also as a powerful tool for dissecting gene functions in plant-pathogen interactions.

### Bacteria stress

Enhancing bacterial resistance in crops using CRISPR/Cas technology involves targeting bacterial virulence genes and enhancing the plant’s immune response by disrupting key genes in bacterial pathogens to significantly reduce their virulence. For instance, knocking out the *StNRL1* gene in potatoes using CRISPR/Cas9 increases resistance to late blight caused by *Phytophthora infestans* while simultaneously increasing susceptibility to early blight caused by *Alternaria alternate* (Norouzi et al., 2024). CRISPR/Cas technology has also been employed to modify plant immune receptors to recognize bacterial pathogens more effectively. Editing the *FERONI* and *SlWak1* depend on *FLS* gene in rice and wheat, which encodes a receptor involved in pathogen recognition, has improved the plants’ ability to detect and respond to bacterial infections, thereby enhancing resistance (Huang et al., 2020; Zhang et al., 2020). This genetic modification has resulted in rice varieties with enhanced resistance to bacterial blight, leading to healthier plants and higher yields.

### Fungi stress

Fungal diseases are a major concern for crop health, and CRISPR/Cas technology offers new ways for enhancing fungal resistance in crops through precise genetic modifications. One strategy involves using CRISPR/Cas to knock out susceptibility genes that fungi exploit, such as *MLO* (Mildew Locus O) genes in soybean and wheat, which has been shown to confer resistance to powdery mildew by making the plants less susceptible to fungal infections (Li et al., 2022a; Bui et al., 2023). Enhancing plant defense genes involved in recognizing and responding to fungal attacks also improves resistance. For instance, editing the *CNL* (Coiled-Coil, Nucleotide-Binding, Leucine-Rich Repeat) gene family and *MeRPPL1* in cassava has led to enhanced fungal resistance (Ramulifho and Rey, 2024). Another innovative application of CRISPR/Cas involves editing the genomes of fungal pathogens themselves. This approach has been explored in various fungal species, including *Fusarium* and *Botrytis*, which are responsible for significant agricultural losses.

### Pests stress

CRISPR/Cas technology is also being utilized to enhance pest resistance in crops by knocking out susceptibility genes and enhancing plant defense mechanisms. For example, editing the ABC transporter gene in soybean has been shown to confer resistance to bollworms by disrupting the insect’s ability to digest plant tissues (Amezian et al., 2024).Another strategy is to enhance the expression of plant defense genes involved in producing secondary metabolites that deter insect feeding. For instance, increasing the expression of genes involved in the biosynthesis of phenolic compounds has been shown to reduce insect herbivory in crops like maize and soybean (Razzaq et al., 2023; Kumari et al., 2024). CRISPR/Cas technology has also been used to modify genes encoding insecticidal proteins, such as *Cry* proteins, *VIP* proteins improving pest resistance in crops (Dubovskiy et al., 2024). These genetic modifications result in plants that produce higher levels of natural insecticidal compounds, providing an effective defense against pests.

### Nematode resistance

Nematodes, such as root-knot nematodes (Meloidogyne spp.), cause root damage and reduce nutrient and water uptake. CRISPR/Cas technology can enhance nematode resistance by targeting genes that facilitate nematode infection and reproduction. For instance, editing a susceptibility gene *OsHPP04* in rice has conferred resistance to root-knot nematodes (Huang et al., 2023). Similarly, modifying the *GmSNAP02* and an *α-SNAP* gene in soybean has enhanced resistance to cyst nematodes (Usovsky et al., 2023, Usovsky et al., 2023). Through disrupting the molecular pathways that nematodes exploit, CRISPR/Cas can develop crops with robust nematode resistance, reducing yield losses and the need for chemical nematicides.

### Parasitic plants

Parasitic plants, such as Striga and Orobanche, attach to the roots of host plants and extract water and nutrients, significantly reducing crop yields. CRISPR/Cas technology can enhance resistance to parasitic plants by targeting genes involved in host-parasite interactions. For instance, editing the *LGS1* gene in sorghum has conferred resistance to *Striga* by disrupting the production of strigolactones, which are essential for Striga seed germination and attachment (Makaza et al., 2023). By modifying specific signaling pathways and defense mechanisms, CRISPR/Cas can develop crops less susceptible to parasitic plants, thereby improving yield and sustainability, while highlighting the technology’s versatility in managing various biotic stresses to ensure better crop health and productivity.

## Yield and quality improvement

Improving crop yield and quality is essential to meet growing global food demand, and CRISPR/Cas technology provides precise tools for enhancing these traits by targeting specific genes and pathways, as explored in this section (**Table 3**).

**Table 3:** Comprehensive overview of CRISPR/Cas applications in enhancing yield, quality, and nutritional value of stable crops.

Application	Type	Innovation	Stable Crops	Targeted genes	Applications in Stable Crops	References
Yield Improvement	Hormone Regulation	CRISPR/Cas9	Rice	*OsCKX*,	Enhance growth and stress tolerance.	(Zheng et al., 2023)
			Maize	*RZ2MS9*		(Figueredo et al., 2023)
	**Photosynthetic Efficiency**		Rice	*RDD*	Suppress miR166 recognition influences photosynthesis	(Iwamoto, 2022)
	**Nutrient Uptake and Utilization**:		Rice	*OsHHO3*	Modify genes involved in nutrient uptake and assimilation.	(Liu et al., 2023a)
			Maize	*ZeSWEET1b*		(Wu et al., 2023)
			Wheat	*ARE1*		(Zhang et al., 2021)
	Yield-Related Traits		Wheat	*TaRPK1*	Influence yield components	(Abdul Rahim et al., 2024)
			Rice	*OsGS2/GRF4*	Increase size and yield	(Wang et al., 2022)
			Barley	*GW2.1*	Reduce seed setting and yield	(Kis et al., 2024)
Quality improvement	**Biofortification**		Cassava	*PSY*	Increase the nutritional content of crops by enhancing vitamin and mineral levels.	(Narayanan et al., 2022)
			Wheat			
			Rice			
	Taste		Maize	*Zmbadh2a, Zmbadh2b*	Improve sugar and acid metabolism	(Wang et al., 2021)
			Rice	*OsBADH2*	Produce a better fragrance	(Imran et al., 2023; Tian et al., 2023)
			Cassava	*CYP79D1*	Lower levels of cyanide	(Juma et al., 2022)
			Potato	*GBSSI*	Obtain amylose-free starch in tubers	(Toinga-Villafuerte et al., 2022)
	Shelf Life		Wheat	*TaPDI*	Accumulate storage protein.	(Hu et al., 2022)
	Texture		Rice	*SD1, Wx*	Enhance semi-dwarf glutinous traits	(Wang et al., 2024d)
			Barley	*Hina*	Increase grain hardness and reduce grain width	(Jiang et al., 2022)
			Potato	*FtsZ1*	Alterate starch granule size in tubers	(Pfotenhauer et al., 2023)
Nutritional Enhancements	Vitamin		Rice	*CRTL, PSY*	Enhance vitamin A content to combat deficiencies.	(Dong et al., 2020)
			Maize			
			Barley	*HGGT, HPT*	Increase vitamin E biosynthesis	(Zeng et al., 2020)
	Amino Acids		Soybean	*GmFAD2*,	Increase fatty acid	(Zhou et al., 2023a)
			Rice	*OsAUX5, OsWRKY78*	Control grain essential amino acid accumulation	(Shi et al., 2023)
	Mineral Content		Rice	*OsNAS2*	Increases Zn uptake/translocation	(Ludwig et al., 2024)
			Wheat	*TalPK1*	Biofortification to increase iron and zinc content.	(Ibrahim et al., 2022)

### Increasing crop yield

CRISPR/Cas technology offers new opportunities to enhance crop yield by directly targeting genes that regulate plant growth and development. For example, editing the *OsAPL* involved in nutrient transport has been shown to increase yield in rice (Zhang et al., 2024a). Enhancing photosynthetic efficiency by targeting genes involved in chlorophyll synthesis and light capture, such as the *OsSXK1* gene in rice, has improved photosynthetic rates and increased grain yield (Zheng et al., 2021). Additionally, editing genes involved in nutrient uptake and utilization, such as the ARE genes in barley or wheat, enhances nitrogen use efficiency and leads to higher yields under low nitrogen conditions (Karunarathne et al., 2022). Recent studies have demonstrated the potential of CRISPR/Cas technology in enhancing yield-related traits in various crops. Editing the *DEP1* gene in rice has led to the development of semi-dwarf varieties with improved lodging resistance and higher grain yield (Zhang et al., 2023a).

### Improving crop quality

CRISPR/Cas9 technology has dramatically advanced agricultural biotechnology by enabling precise genome editing to improve various crop quality attributes, including safety, taste, texture, shelf life, and industrial applicability. In cassava, CRISPR/Cas9 has been used to edit the *CYP79D1* gene, significantly reducing cyanogenic glycosides, which lowers the risk of cyanide toxicity, enhancing the safety of this staple crop without affecting its agronomic performance (Juma et al., 2022). In rice, the technology has been employed to enhance aromatic qualities by editing the *OsBADH2* gene, leading to increased production of 2-acetyl-1-pyrroline (2-AP), a compound that imparts a desirable fragrance, thereby catering to consumer preferences (Tian et al., 2023). In potatoes, CRISPR/Cas9 has modified the *gbss* gene responsible for granule-bound starch synthase, resulting in amylose-free starch that provides a smoother texture, which is highly valued in both culinary applications and industrial processes (Toinga-Villafuerte et al., 2022). The technology has also been pivotal in extending the shelf life of various crops by targeting genes involved in the ripening process, such as those regulating ethylene production, allowing for slower ripening, reduced post-harvest losses, and improved economic viability. Furthermore, in barley, CRISPR/Cas9 has been used to enhance grain hardness by editing the *Hina* gene, producing grains with a higher hardness index that are better suited for industrial applications, though this has also resulted in reduced grain width and thousand-grain weight (Jiang et al., 2022). Additionally, in potatoes, targeting the *FtsZ1* gene has led to the development of lines with larger starch granules, significantly increasing the final viscosity of starch paste, making these potatoes more suitable for specific industrial processes, all achieved without compromising the plant’s overall phenotype or nutritional quality (Pfotenhauer et al., 2023). These diverse applications of CRISPR/Cas9 underscore its transformative potential in crop improvement, enabling tailored modifications that meet consumer demands, enhance safety, and address specific industrial needs while ensuring the sustainability and economic viability of agricultural practices.

### Nutritional enhancements

Addressing nutritional deficiencies through crop biofortification is a key goal in agricultural biotechnology, and CRISPR/Cas technology plays a pivotal role in achieving this. For instance, CRISPR/Cas has been employed to increase pro-vitamin A content in rice, a vital intervention in combating vitamin A deficiency in populations that rely heavily on rice as a staple food (Maiti and Banik, 2023). Biofortification aims to increase the content of essential nutrients in crops, thereby improving their nutritional value. The development of “Golden Rice,” which contains higher levels of beta-carotene, was accomplished by modifying genes involved in pro-vitamin A biosynthesis (Dong et al., 2020; Datta et al., 2021). RISPR/Cas technology has also been applied to enhance the mineral content of crops. In rice and wheat, genes such as *OsNAS* have been edited to increase iron and zinc levels, addressing micronutrient deficiencies that often lead to anemia and impaired immune function (Dueñas et al., 2021). Similarly, in maize, targeting the *PSY1*, *Crtl*, and *LCYB* genes has boosted the biosynthesis of pro-vitamin A, resulting in the creation of “Golden Maize” (Sobrino-Mengual et al., 2024).The technology is also instrumental in improving the amino acid content of crops. In cassava, CRISPR/Cas has been used to enhance the levels of essential amino acids and vitamins, significantly improving the nutritional quality of this staple crop (Otun et al., 2023). In maize, editing genes involved in lysine biosynthesis has increased lysine content, addressing a common deficiency in cereal grains (Hasan, 2024). Improving the protein quality of crops is another significant area of focus for CRISPR/Cas technology. For example, targeted mutagenesis of the *OsAAP6* and *OsAAP10* genes in rice can reduce grain protein content, thereby improving the eating and cooking quality of the crop (Wang et al., 2020). CRISPR/Cas technology has been used to reduce antinutritional factors such as phytic acid in soybean by targeting the *GmIPK1* gene, enhancing the bioavailability of iron and zinc and thereby improving the overall nutritional quality of the soybean (Song et al., 2022).

## Cases study of applications in staple crops

**Figure 4:**
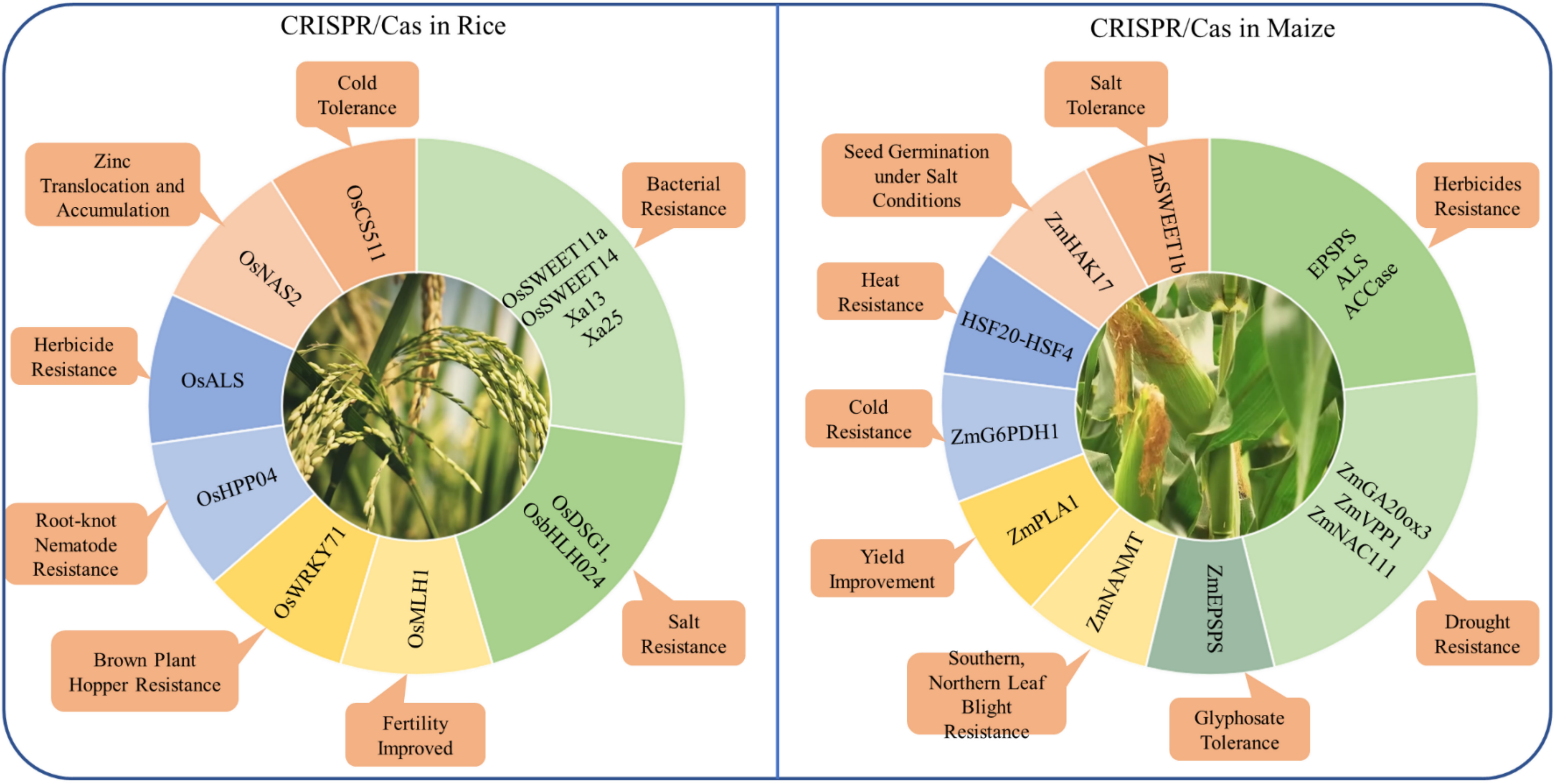
Application of CRISPR/Cas genome editing in rice and maize for the improvement of different traits.

As the application of CRISPR/Cas technology is broad and impactful across various staple crops, focusing on specific case studies such as rice and maize allows us to delve deeper into its transformative role in enhancing resilience (**Figure 4**).

### Case study: CRISPR/Cas in rice

CRISPR/Cas technology has significantly advanced rice improvement by enabling precise genome modifications, enhancing traits such as yield, stress resistance, and nutritional value. This technology has been instrumental in improving resistance to both biotic and abiotic stresses in rice. For example, the clade III subfamily of *OsSWEETs*, including *OsSWEET11a* and *OsSWEET14*, plays a crucial role in susceptibility to bacterial blight by mediating sucrose efflux for bacterial proliferation. These genes are targeted by specific transcription activator-like effectors (*TALEs*) from *Xoo* (Wang et al., 2024g). This approach demonstrates the potential of CRISPR/Cas9 to address other pathogen-related challenges in rice. Similarly, the *OsCS511* gene has been edited to improve cold tolerance in rice, which is critical for expanding rice cultivation to cooler climates (Park et al., 2024). It highlights the adaptability of CRISPR/Cas technology in enabling rice to grow in suboptimal conditions, thereby expanding its cultivation area. Furthermore, base editing has been used to increase zinc uptake and plant yield by editing the promoter region of the *OsNAS2* gene, which plays a crucial role in zinc translocation and accumulation (Ludwig et al., 2024). This precise editing has led to a significant increase in zinc concentration in rice grains, addressing both yield improvement and nutritional enhancement in a single genetic modification. Additionally, the knockout of the *OsDSG1* and *OsbHLH024* transcription factor has been shown to significantly enhance salt stress resistance, allowing rice to thrive in saline soils (Alam et al., 2022; Ly et al., 2024). This advancement is particularly important in regions where soil salinity limits agricultural productivity. Furthermore, CRISPR/Cas9-mediated mutagenesis of the susceptibility gene *OsHPP04* has conferred enhanced resistance to rice root-knot nematode, a significant pest that causes major yield losses in rice crops, demonstrating CRISPR/Cas9’s potential in sustainable rice production (Huang et al., 2023).

Despite these successes, challenges such as off-target effects, potentially lead to undesirable mutations. For instance, when editing the *OsWRKY71* gene to enhance resistance against brown plant hopper, careful analysis was necessary to ensure that no off-target effects compromised the plant’s health (Li et al., 2023b). This case underscores the importance of precision in gene editing to avoid unintended consequences that could negate the benefits of the modifications. Another challenge is the relatively low efficiency of HDR in rice, which is required for precise gene insertions or complex modifications. This limitation has been addressed in part by developing more precise methods like prime editing, which does not rely on HDR. Prime editing has been successfully applied to the *OsMLH1* gene to enhance the efficiency of the editing process without disturbing rice fertility, offering a promising approach for future genetic improvements (Liu et al., 2024a).

Recent advancements in CRISPR technology, particularly base editing and prime editing, have opened new avenues for improving specific traits in rice. Base editing has been successfully applied to the *OsALS* gene to confer herbicide resistance, allowing rice to resistant to herbicides such as imazamox, which is crucial for effective weed management (Zafar et al., 2023). This modification allows for more targeted and sustainable weed control strategies, reducing the need for broad-spectrum herbicides that can harm the environment. Additionally, prime editing has been applied to the *Xa13* and *Xa25* genes, creating rice varieties with enhanced resistance to bacterial blight by targeting and disrupting these susceptibility genes (Zhu et al., 2024). These innovations, including the application of base editing and prime editing to improve traits like herbicide resistance and bacterial blight resistance, are crucial for developing resilient rice varieties suited to climate change and global food security challenges.

### Case study: CRISPR/Cas in maize

The application of CRISPR/Cas systems, including advanced techniques like prime editing and base editing, has revolutionized the ability to enhance maize’s tolerance to various abiotic and biotic stresses. Prime editing, particularly the optimized ePE5max system, has been instrumental in generating heritable mutations in maize, enabling resistance to herbicides by targeting key enzymes such as *EPSPS*, *ALS*, and *ACCase*. This has significantly improved the resilience of maize to herbicide stress. Base editing has also shown potential in this area, with the introduction of specific nucleotide substitutions, such as the triple amino acid substitution in the *ZmEPSPS* gene, leading to heightened glyphosate tolerance (Kaul et al., 2024). Additionally, the editing of *ZmSWEET1b*, a sugar transporter crucial for assimilate allocation, has demonstrated improved salt stress response, showcasing the effectiveness of these gene-editing techniques in enhancing maize’s abiotic stress tolerance (Wu et al., 2023).

Further advancements in maize stress tolerance have been achieved through the targeted editing of specific genes associated with drought, salinity, and disease resistance. For example, the gene editing of *ZmGA20ox3* has not only improved drought tolerance but also optimized plant architecture, while the co-expression of *ZmVPP1* with *ZmNAC111* has conferred robust drought resistance by enhancing the plant’s stress response mechanisms (Liu et al., 2023d; Liu et al., 2024b). Similarly, genes like *ZmHAK17*, which encodes a Na^+^-selective transporter, and the *HKT1* family sodium transporter have been pivotal in improving maize’s tolerance to salinity by regulating sodium influx and promoting seed germination under salt conditions (Zhang et al., 2023d; Wang et al., 2024a). Moreover, the genome editing of the *ZmNANMT* gene has conferred multiple disease resistance without agronomic penalties, addressing major diseases like southern leaf blight, northern leaf blight, and *Fusarium* stalk rot (Li et al., 2023c). These targeted genetic modifications underline the versatility of CRISPR/Cas systems in addressing both biotic and abiotic stresses in maize.

However, despite these promising developments, the application of CRISPR/Cas systems in maize faces several challenges, including off-target effects, regulatory hurdles, and efficiency in gene editing techniques. For instance, while the DNA-free genome editing of the *ZmPLA1* gene via ribonucleoprotein complexes has been successful in increasing haploid induction rates in tropical maize, optimizing these methods further remains crucial to enhancing efficiency and reducing unintended mutations (Rangari et al., 2023). Similarly, the generation of maize lines with enhanced cold stress tolerance through the overexpression of *ZmG6PDH1* in glucose-6-phosphate dehydrogenase family, and the regulation of heat stress tolerance through the *HSF20-HSF4*-cellulose synthase A2 module, illustrate the complexity of stress responses in maize that require precise and efficient gene editing tools (Li et al., 2023a, Li et al., 2024b). Addressing these challenges through ongoing research and technological advancements will be key to fully harnessing the potential of CRISPR/Cas systems in improving maize resilience and productivity under various environmental stresses.

## Challenges and future prospect

### Off-target effects

Off-target effects in CRISPR/Cas systems present a significant challenge in genome editing, where unintended cuts by the Cas enzyme at non-target sites can lead to adverse consequences. In crops, such unintended mutations can affect traits, reducing yield or introducing unwanted characteristics. Therefore, minimizing off-target effects is critical to ensuring the safety and efficacy of CRISPR-based technologies, making precision in genome editing a top priority. To address these challenges, considerable progress has been made in developing high-fidelity Cas variants and optimizing gRNA design. High-fidelity Cas9 variants, such as eSpCas9, SpCas9-HF1 and FrCas9, have been engineered to enhance precision by reducing nonspecific DNA interactions (Guo et al., 2023; He et al., 2024). These variants demonstrate lower off-target activity while maintaining robust on-target efficacy, making them suitable for applications. Additionally, studies have shown that Cas12b, with its strict PAM requirements and low tolerance for mismatches, has minimal sgRNA-dependent off-target effects, showing great promise in rice genome editing (Gurel et al., 2023). This system’s ability to precisely target specific genomic sites with minimal off-target activity underscores the importance of optimizing both the Cas enzyme and gRNA design to enhance the specificity of CRISPR/Cas systems. Future research should focus on enhancing accuracy, minimizing off-target effects, and developing novel variants or engineered Cas proteins that can better handle the complexities of multiplex editing, especially in crops with polyploid genomes. This will not only improve precision in genome editing but also reduce unintended consequences, making CRISPR technology safer and more reliable for agricultural applications.

### Delivery methods

Delivering CRISPR components into plant cells, particularly in recalcitrant staple crops like wheat, maize, and certain rice varieties, presents significant challenges due to their complex genetic makeup and poor tissue culture responses. Traditional methods, such as Agrobacterium-mediated transformation and biolistic particle delivery, are often ineffective in these crops. Agrobacterium-mediated transformation is limited by its host range and often results in random transgene integration, leading to gene silencing and unpredictable expression patterns. Similarly, while biolistic delivery bypasses some limitations of Agrobacterium, it frequently causes multiple, unstable transgene insertions and physical damage to plant tissues, reducing transformation efficiency. Nanoparticle-mediated delivery, a more recent approach, shows promise but faces challenges related to the precise release and stability of CRISPR components within plant cells, as well as potential cytotoxicity concerns (Antony Ceasar and Ignacimuthu, 2023).

To overcome these challenges, research is increasingly focused on developing novel delivery methods that enhance the efficiency and specificity of CRISPR delivery in recalcitrant crops (Kocsisova and Coneva, 2023). Viral vectors offer a promising avenue by utilizing natural plant infection mechanisms to deliver CRISPR components, though their limited cargo capacity remains a significant hurdle (Liu et al., 2023c). Enhancements in nanoparticle systems, such as surface functionalization with specific ligands and the development of biodegradable particles, could improve the specificity and safety of CRISPR delivery. Protoplast transfection, combined with optimized regeneration protocols, offers a direct method for introducing CRISPR components, though its application is currently limited to species with efficient protoplast regeneration systems (Yang et al., 2024). Grafting techniques, which use transgenic rootstocks to deliver CRISPR components to wild-type scions, represent another innovative approach for achieving transgene-free genome editing in recalcitrant crops (Yang et al., 2023b). To achieve sustainable improvements, future research should not only focus on developing novel delivery methods but also on ensuring the long-term stability and heritability of CRISPR-induced modifications across multiple generations. Understanding mechanisms such as chromosomal rearrangements and DNA repair fidelity will be crucial to maintaining the integrity of these genetic edits. Longitudinal studies that track the persistence and effects of these modifications over time are essential to confirm their stability and effectiveness, ensuring that the benefits of CRISPR technology are retained through successive crop generations.

### Ethical, and regulatory issue and public acceptance

CRISPR technology offers significant potential in crop improvement, but it also presents challenges in ethics, regulation, and socioeconomic impact. Ethically, the precision and speed of CRISPR modifications, such as those used in rice and wheat to enhance yield and nutritional content, raise concerns about the moral acceptability of altering plant genomes. These concerns are especially pronounced in regions like Europe, where public sentiment is cautious about genetic modifications. The ethical debate includes potential unintended consequences, the creation of “unnatural” organisms, and the long-term ecological impacts (Marone et al., 2023). Additionally, the regulatory landscape for CRISPR-edited crops varies significantly across countries. In European Union classifies CRISPR-edited crops as genetically modified organisms (GMOs), subjecting them to rigorous regulations that have hindered the commercialization of crops like CRISPR-edited wheat. Countries like Argentina and Brazil have adopted more flexible regulatory frameworks, focusing on the final product rather than the process, allowing for the development and commercialization of crops like CRISPR-edited sugarcane with less regulatory burden. Meanwhile, China’s significant investment in CRISPR research, particularly in crops like rice, positions it as a major player, although commercialization remains tightly regulated (Ghouri et al., 2023; Kumawat et al., 2024). Socioeconomic challenges further complicate the integration of CRISPR technology into global agriculture. The contentious patent landscape, dominated by the U.S. and European nations, restricts access to CRISPR technology, particularly for smaller entities or developing countries. This is evident in the development of CRISPR-edited crops like tomatoes with enhanced GABA levels, where patent issues could limit access in less affluent regions (Akhtar et al., 2023). Additionally, the concentration of CRISPR technology within a few large corporations could exacerbate inequalities in the agricultural sector, particularly for staple crops like maize and wheat, crucial for food security in developing regions (Molinari et al., 2024). Countries like India and several African nations face the challenge of ensuring that the benefits of CRISPR technology, such as drought-resistant maize or disease-resistant cassava, are accessible to all farmers, not just large agribusinesses (Munawar et al., 2024). As CRISPR technology advances, ensuring that regulatory frameworks keep pace with these developments is essential. Future research must focus on addressing safety concerns, including off-target effects and the long-term ecological impacts of CRISPR-modified crops. This requires the development of comprehensive risk assessment models and strong collaboration between scientists, regulators, and policymakers. By aligning technological advancements with robust regulatory measures, we can ensure the safe and responsible integration of CRISPR into global agricultural practices, ultimately fostering public trust and acceptance.

### Synergy of CRISPR with advancement technologies

The intersection of CRISPR technology with emerging fields such as nanotechnology, synthetic biology, and machine learning (ML) offers a transformative potential to advance genome editing in staple crops like rice, maize, wheat, and potato. Nanotechnology, in particular, addresses one of the critical challenges in CRISPR-based genome editing: the efficient and precise delivery of CRISPR components into plant cells. Nanomaterials like carbon nanotubes and mesoporous silica nanoparticles can bypass the plant cell wall, enabling targeted and controlled delivery of CRISPR components, which increases transformation efficiency and reduces off-target effects (Khanna et al., 2023). Additionally, nanoparticle-mediated delivery systems are species-independent, democratizing CRISPR technology across diverse crops (Naik et al., 2022). Future research should focus on optimizing these nanotechnologies for larger CRISPR complexes, organelle-specific editing, and direct transformation of germline cells, potentially bypassing tissue culture. This synergy holds great potential for advancing sustainable agriculture, improving crop resilience, nutritional content, and reducing chemical input dependency.

The intersection of CRISPR and Synthetic Biology offers a promising path to enhance genome editing efficiency and precision in staple crops. CRISPR/Cas systems enable targeted modifications, but challenges such as off-target effects, variable efficiency, and polyploid genome complexity persist. Synthetic Biology addresses these issues by providing tools to design and control genetic circuits, thereby reducing off-target effects and improving adaptability across different species (Yang and Reyna-Llorens, 2023). Key advancements include regulatory circuits that fine-tune CRISPR activity and feedback loops that adjust editing in real-time, enhancing precision (Wang and Demirer, 2023). Integrating CRISPR with synthetic metabolic pathways could yield crops that are higher-yielding and more resilient to environmental stressors, crucial for addressing global food security challenges posed by climate change. Future research should focus on developing synthetic promoters for efficient editing in polyploid crops and combining CRISPR with metabolic engineering to produce bioactive compounds, advancing sustainable agriculture in both traditional and controlled environments like vertical farming.

Machine learning (ML) adds another layer of innovation by exponentially enhancing CRISPR’s potential for precise genome modifications. However, when combined with the predictive capabilities of ML, the potential of CRISPR can be vastly expanded. One of the key challenges in CRISPR genome editing is ensuring specificity and efficiency in targeting the correct genomic sites while minimizing off-target effects. ML models can address this challenge by analyzing large datasets from CRISPR experiments to predict the most effective guide RNA sequences, thereby enhancing the precision of the Cas9 enzyme and reducing unintended consequences (Chen et al., 2024). Additionally, ML can predict the phenotypic outcomes of specific gene edits, which is particularly complex due to the multifactorial nature of traits like yield and stress tolerance (Das et al., 2023). In crops like rice and maize, where multiple genes interact to influence traits such as drought resistance, ML can identify the most impactful gene edits by considering various genetic and environmental factors. Future research should focus on developing sophisticated ML algorithms capable of handling the complexity of polygenic traits and creating extensive datasets to train these models. Integrating CRISPR-ML into precision agriculture systems could provide tailored recommendations, optimizing crop performance in specific field conditions and ultimately contributing to more sustainable and efficient agricultural practices.

### Future applications with CRISPR/Cas knock-in system

While the CRISPR/Cas9 system has predominantly been used for gene knockouts, the development of CRISPR/Cas-based knock-in strategies has significantly expanded its potential, enabling precise gene integration and enhancing crop traits with high accuracy. This system allows for the insertion of desired genes into specific genomic locations, facilitating complex genetic modifications such as the introduction of large DNA sequences or multiple genes, which are crucial for stacking beneficial traits like disease resistance, stress tolerance, and improved nutritional content. However, the knock-in approach relies primarily on HDR, which is less efficient in plants compared to NHEJ. To address this limitation, researchers are developing strategies to enhance HDR efficiency, including the use of HDR enhancers, dual-gRNA systems, and advanced delivery methods like nanoparticle-mediated delivery and viral vectors, making the knock-in process more feasible for large-scale agricultural applications.

The application of the CRISPR/Cas knock-in system in specific crops has demonstrated its versatility and promise in sustainable agriculture. For instance, in rice, this system has been used to confer glyphosate resistance by precisely editing the acetolactate synthase (ALS) gene and to upregulate genes involved in key metabolic pathways, significantly enhancing the crop’s nutritional content and stress tolerance (Sony et al., 2023). In wheat, researchers are focusing on improving HDR efficiency using fusion proteins like Cas9-VirD2 to enhance traits such as disease resistance and grain quality (Schreiber et al., 2024). Similarly, in maize, the knock-in of regulatory elements to more precisely control gene expression has been explored to improve yield, nutrient use efficiency, and stress tolerance (Kaul et al., 2024). These examples underscore the knock-in system’s crucial role in developing next-generation crops that are more resilient, productive, and adaptable to changing environmental conditions.

Despite its potential, the CRISPR/Cas knock-in system faces challenges, including low HDR efficiency and off-target effects. To overcome these, researchers are exploring various strategies, such as enhancing HDR pathways, developing modified CRISPR/Cas variants, and utilizing alternative genome editing tools like CRISPR nickase and prime editing, which reduce the risk of off-target mutations. Improving delivery systems, such as nanoparticle-based methods, is also critical for increasing the efficiency and precision of gene insertion. Future directions for this technology include further engineering of Cas proteins for enhanced specificity, optimizing guide RNA design to minimize off-target effects, and integrating the knock-in system with emerging technologies like base editing and prime editing. These advancements are essential for refining the CRISPR/Cas knock-in system and maximizing its impact on agricultural innovation.

## Conclusion

In conclusion, this review underscores the transformative potential of CRISPR/Cas technology in improving the resilience, yield, and nutritional value of staple crops like rice and maize. Through precise genome modifications, CRISPR/Cas systems have revolutionized crop breeding by enhancing stress tolerance, disease resistance, and overall productivity. Recent advancements, including base editing, prime editing, and high-fidelity Cas variants, have significantly increased the specificity and efficiency of genome editing, reducing off-target effects and expanding its agricultural applications. Furthermore, CRISPR/Cas technology has played a crucial role in biofortification efforts, such as boosting pro-vitamin A content in rice and increasing iron and zinc levels in wheat, addressing critical global challenges like food security and malnutrition, particularly in developing regions. To fully harness the potential of CRISPR/Cas technology, future research should focus on improving HDR efficiency, expanding the CRISPR toolkit, addressing ethical and regulatory challenges, and integrating CRISPR into traditional breeding programs to accelerate the development of high-yielding, climate-resilient crops, thereby contributing to sustainable and resilient global food systems.
